# COVID-19 prevention and control strategies: learning from the Macau model

**DOI:** 10.7150/ijbs.70177

**Published:** 2022-08-21

**Authors:** Yan-Jie Zhao, Jia-Qi Xu, Wei Bai, He-Li Sun, Bing Shui, Zhi-Xin Yang, Jingbo Huang, Robert D. Smith, Yuan-Jia Hu, Yu-Tao Xiang

**Affiliations:** 1 The National Clinical Research Center for Mental Disorders & Beijing Key Laboratory of Mental Disorders, Beijing Anding Hospital & the Advanced Innovation Center for Human Brain Protection, Capital Medical University, Beijing, China.; 2 Unit of Psychiatry, Department of Public Health and Medicinal Administration, & Institute of Translational Medicine, Faculty of Health Sciences, University of Macau, Macau SAR, China.; 3 Centre for Cognitive and Brain Sciences, University of Macau, Macau SAR, China.; 4 Department of Public Health and Medicinal Administration, Institute of Chinese Medical Sciences, University of Macau, Macau SAR, China.; 5 Faculty of Law, University of Macau, Macau SAR, China.; 6 Faculty of Science and Technology, University of Macau, Macau SAR, China.; 7 United Nations University Institute in Macau, Macau SAR, China.

**Keywords:** COVID-19, Macau, strategy

## Abstract

**Background:** Macau is a densely populated international tourist city. Compared to most tensely populated countries/territories, the prevalence and mortality of COVID-19 in Macau are lower. The experiences in Macau could be helpful for other areas to combat the COVID-19 pandemic. This article introduced the endeavours and achievements of Macau in combatting the COVID-19 pandemic.

**Method:** Both qualitative and quantitative analysis methods were used to explore the work, measures, and achievements of Macau in dealing with the COVID-19 pandemic.

**Results:** The results revealed that Macau has provided undifferentiated mask purchase reservation services, COVID-19 vaccination services to all residents and non-residents in Macau along with delivering multilingual services, in Chinese, English and Portuguese, to different groups of the population. To facilitate the travels of people, business and trades between Macau and mainland China, the Macau government launched the Macau Health Code System, which uses the health status declaration, residence history declaration, contact history declaration of the declarant to match various relevant backend databases within the health authority and provide a risk-related colour code operations. The Macau Health Code System connects to the Chinese mainland's own propriety health code system seamlessly, whilst effectively protecting the privacy of the residents. Macau has also developed the COVID-19 Vaccination Appointment system, the Nucleic Acid Test Appointment system, the Port and Entry/Exit Quarantine system, the medical and other supporting systems.

**Conclusion:** The efforts in Macau have achieved remarkable results in COVID-19 prevention and control, effectively safeguarding the lives and health of the people and manifesting the core principle of “serving the public”. The measures used are sustainable and can serve as an important reference for other countries/regions.

## Introduction

The outbreak of coronavirus disease 2019 (COVID-19) was first reported in Hubei province of China at the end of 2019, and subsequently also emerged in more than 200 countries and territories 1, 2. The World Health Organization (WHO) declared that COVID-19 can be characterized as a pandemic 3. As of August 2022, there were over 587 million COVID-19 cases globally with over 6.4 million deaths 4.

Compared to most parts of the world, the COVID-19 strategy in Macau is more successful. As of 11 August 2022, there were only 791 confirmed cases, with 6 death cases 5. Macau is the most densely populated international tourist city in the world with a population density of 20,700 people per square kilometre 6, and annual visitors approaching 40 million before the COVID-19 pandemic 7. The experiences in Macau could be helpful for other densely populated countries/territories to combat the COVID-19 pandemic.

The purpose of this article is to introduce the endeavours, results, and achievements of the Macau SAR government in the management of the COVID-19 pandemic. This report focused on several aspects, including technological innovations, significant achievements in COVID-19 pandemic management, and adaptability to other countries/regions, sustainability, and coordination and partnership with non-governmental bodies.

## Materials and Methods

Both qualitative and quantitative research methods were used to examine the work, measures, and results of the Macau SAR government in dealing with the COVID-19 pandemic.

Our qualitative research involves reviewing, evaluating, and studying written materials of the period from the outbreak of the COVID-19 at the end of 2019 to the middle of 2022, such as the public information, the databases on population and economy published by the government, the government's policy addresses, news reports, interviews, and published literature.

Quantitative research involves a secondary analysis of existing open databases of the government, using descriptive analysis and comparative analysis methods to describe the changes in the number of inbound visitors of Macau and other control regions, in order to reflect the progress of the local economic recovery.

## Results

### Management of the COVID-19 pandemic

#### Disease control and prevention

The Macau SAR Government attaches great importance to the prevention and control of infectious diseases. Upon learning of the outbreak of the pneumonia of unknown origin, the Health Bureau immediately dispatched personnel equipped with protective gear to carry out quarantine measures on board of aircrafts on 1 January 2020 8, 9. The measures included taking the body temperature of all passengers in the cabin of flights from Wuhan, requiring all inbound travelers to fill in a health declaration form, stepping up fever screening at all ports of entry, establishing and improving the monitoring and checking plans within a short time, and suspending the tour groups between Macau and Wuhan 9, 10.

Following the first case confirmed in Macau on 22 January 2020 11, the authority announced a series of preventive measures, which included ordering masks for local residents to purchase to ensure that every resident had access to one mask to use per day, postponing the resumption of classes after the Spring Festival holiday in local primary and secondary schools to prevent risk of students from becoming infected on campus, authorizing border guards to prevent passengers with fever from crossing the border, and even closing casinos when deemed necessary 12. The health declaration and fever screening measures were fully implemented at all the ports of entry from 23 January 2020 13. Additionally, a team that consisted of public health experts was also set up, which proposed ideas such as case tracing, the Guaranteed Mask Supply Scheme, protection of vulnerable populations, and health services' education for the public, providing scientific guidance and an important theoretical basis for the management of zero-COVID policy in Macau.

Among the measures taken by the Macau SAR Government in combating COVID-19, the Guaranteed Mask Supply Scheme plays an effective role. A meta-analysis of 172 observational studies from 16 countries found that wearing a mask significantly reduced the risk of infection (n=2,647; adjusted OR=0.15, 95% CI: 0.07~0.34) 14. To effectively prevent the spread of the virus, the Macau SAR Government announced the Guaranteed Mask Supply Scheme at the press conference held on the afternoon of 22 January 2020 and started implementing the scheme (real-name mask purchase) on 23 January 2020 15. The scheme allows Macau residents and non-resident workers to purchase masks from contracted pharmacies at a cost of MOP 8 (approximately one US dollar) for 10 masks every 10 days 15. This ensures that every resident was able to wear a new mask each day. The Macau SAR Government even purchased masks from suppliers worldwide at great cost to ensure the supply of masks in order to cut off the transmission channels, protect the community, and safeguard social stability and people's confidence.

**Figure 1:**
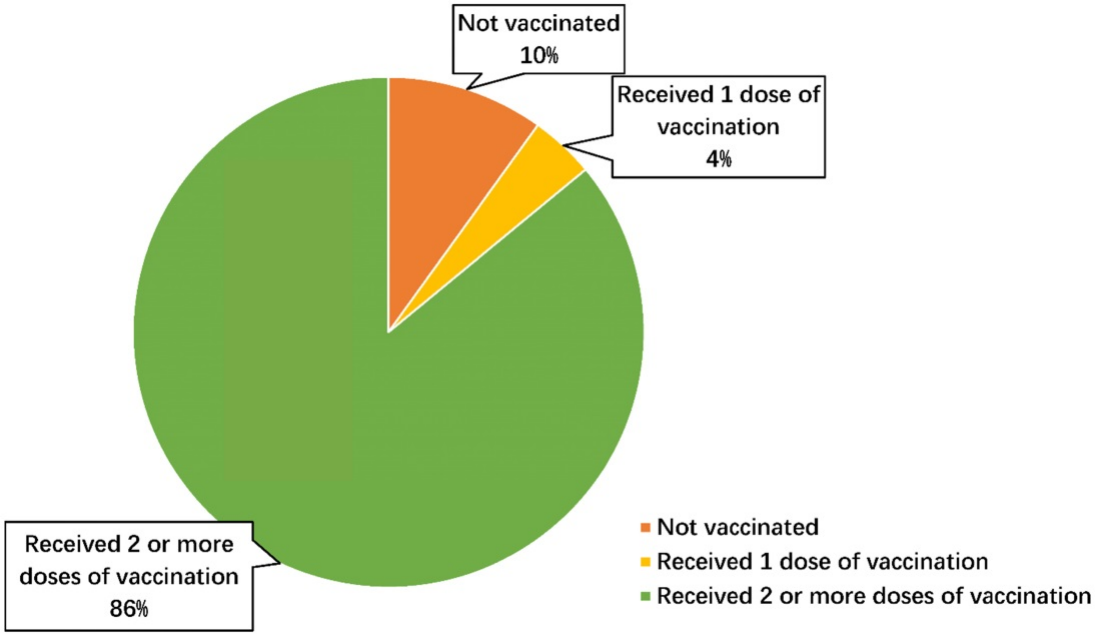
The COVID-19 vaccination rate in Macau (Data source: Reference 5)

The Macau SAR government has encouraged the public to actively participate in vaccination to build an immune barrier. The Health Bureau launched the COVID-19 Vaccination system on 9 February 2021, offering different vaccines for the public to choose from 16. The first phase of vaccination is open to priority groups, including people working at the front line of the pandemic prevention and control, people with high occupational exposure, and people who urgently need to travel to high-risk countries or regions. The second phase is extended to all Macau residents, with appointments starting at 12 noon on 9 February 2021 for vaccination dates on or after 22 February 2021 16, 17. The third phase is open to non-resident workers, with appointments starting on 9 March 2021 and vaccination starting from 10 March 2021. No-local students studying in Macau and other non-residents who are legally permitted to stay in Macau (those who have stayed legally in Macau for more than half of the past 6 months) could make appointments for vaccination from 10 a.m. on 9 April 2021 and can receive the inactivated vaccine (Sinopharm CNBG Beijing) on 10 April and the mRNA vaccine (BioNTech) on 12 April at the earliest 18. As of August 11, 2022, 1.46 million doses of vaccines have been administered in Macau SAR, with a total vaccination rate of 90% 5. The COVID-19 vaccination rate in Macau is shown in Figure 1.

#### Financial aspects

In response to the sudden and dramatic outbreak of the pandemic, the Macau SAR government has launched several rounds of general and targeted financial support programs in two successive phases of efforts towards pandemic control and economic recovery, attaining considerable success in relieving people's hardships, stabilising the economy, securing local employment, while the entire society has remained stable.

##### Enhancing market monitoring to ensure the stability of food supplies

The Economic and Technological Development Bureau and the Consumer Council conducted on-site investigations and the results showed that the supply and inventory of all daily necessities have been sufficient, their prices stable, and the supply chain of essential goods functioning smoothly amid the pandemic. In addition, a total of 264 branches of the 16 supermarkets, department stores, and retailing groups in Macau have worked all out to ensure supply stability and maintain order in purchases and replenish goods as soon as possible in line with the sales and demand situation and, at the same time, have taken all pandemic prevention measures in accordance with the guidelines of the health authority. Moreover, in a bid to increase price transparency, maintain a balance between supply and demand, and protect consumer rights and interests, the Consumer Council launched the “Macau Price Information Platform”, through which users would be able to check the instant price information at their convenience 19, 20.

##### Implementing tax reduction and waiver initiatives to sustain businesses

The SAR government has launched a set of proactive policies such as assistance business financing, rental waivers at government-owned properties, tax facilitation packages, and other support programs with the goal of helping business operators and employees to tide over the difficulties, protecting local employment and maintaining social stability.

The tax reduction and exemption measures, with a total amount of MOP 1.056 billion 21, included: adjusting the complementary income tax, with a deducting of the tax collection in 2019 up to MOP300,000 for commercial SMEs, around 2,970 companies in total; adjusting the professional tax, with 70 per cent of the professional tax collection in 2018 to be returned up to a maximum limit of MOP20,000 (2,467.6 USD), benefiting 170,000 local employees; increasing the fixed deduction in the taxable income of professional tax in 2020 from 25% to 30%, benefiting 180,000 local employees; exempting all housing taxes levied on Macau residents' residences in 2020, benefiting 180,000 families; exempting 25 per cent of the housing tax on commercial premises, benefiting 25,000 commercial establishments; exempting tourism tax on tourism service venues such as hotels, bars, gymnasiums and karaoke bars for a period of 6 months, benefiting 854 establishments; rebating the license tax for all business vehicles; exempting or rebating administrative license fees and stamp duty levied by various administrative departments and entities in 2020 22.

The SAR government also implemented the support and interest-subsidy scheme for small, medium, and micro enterprises to support their operation. A provisional credit interest subsidy scheme for SMEs was launched, targeting the SMEs that have been granted loans by banks due to their shortage of funds caused by the pandemic. The government gave an interest subsidy of 4% for loans up to a maximum amount of MOP 2 million for a maximum period of 3 years. In addition, the SAR government also launched a special support scheme for SMEs, providing eligible SMEs with an interest-free loan to a maximum of MOP 600,000 (74,027.7 USD) 22.

##### Local consumption promotion plan to boost domestic demand

The local consumption promotion plan is threefold, which includes the electronic consumption discounts, the elderly consumption discounts, and the local tour programs for meals, accommodation and excursions 23. Through the Wealth Partaking Scheme, the SAR government spent a total amount of MOP 7.235 billion (892.65 million USD) in cash handout distribution, with each permanent resident of Macau receiving MOP10,000 (1,233.8 USD), and each non-permanent resident receiving MOP6,000 (740.3 USD). In addition, a total of MOP 5 billion (616.9 million USD) was injected into the local market through the electronic voucher scheme. Macau residents, non-resident workers and non-resident tertiary students could all enjoy the e-voucher discount via e-payment apps on their mobile phones after completing real-name verification. The elderly residents who are not familiar with the mode of electronic mobile payment have also been taken into consideration, while Macau residents aged 65 or above have been provided with the Senior Card to enjoy discounts in their consumption of up to MOP5,000 (616.9 USD). Moreover, Macau residents have been encouraged to participate in local tours in a bid to boost local consumption and support the tourism industry. In the “Macau Ready Go! Local Tours” program for local itineraries, each local resident who joins would receive a subsidy of MOP280 (34.5 USD) for travel and a one-off MOP200 (24.7 USD) subsidy for hotel accommodation.

##### Other initiatives to benefit residents' livelihood

The “Assistance Payment Plan for Employees, Freelancers and Business Operators” was endorsed for distributing assistance payments of various amounts to eligible employees, freelancers who do not hire employees, and business operators 24. In addition, targeted employment support schemes were implemented for residents, including the “Employment Oriented Training Program with Subsidy” for the unemployed residents and higher education graduates, and the “Skill-Enhancing Oriented Training Program with Subsidy” for incumbent employees. A total amount of MOP334 (41.2 USD) million was invested with a view to assisting employees to upgrade their vocational skills, and the training programs with subsidies have been optimised. The “Special Healthcare Support Program” was implemented, which provided Macau residents with an additional MOP600 (74 USD) health voucher to be spent on their personal health and to support anti-pandemic efforts 25. Other welfare measures include subsidies for all electricity and water bills for the housing units of Macau residents up to a period of 3 months, and an additional two month-worth of subsidies to that disadvantaged families that are receiving subsidies from the Social Welfare Bureau 26.

##### Implementing proactive fiscal policies by increasing public investment

During the time when the pandemic situation was stable, the SAR government was actively engaged in promoting adequate economic diversification and growth, and, according to the actual situation, building an adequately diversified industrial structure for sustainable development. The SAR government has worked actively to foster and develop a “big health” industry, taking the research and development and manufacturing of traditional Chinese medicine (TCM) as an entry point, while facilitating more proprietary TCM products to be registered in Macau and to gradually enter the mainland market. In addition, modern financial services have been actively boosted for development, a bond market has been developed at an accelerated pace, and wealth management and financial leasing services have been vigorously promoted. Efforts have also been made for building a cross-border RMB settlement centre, and expanding green finance services. To this end, the construction of financial infrastructure has been strengthened, and the financial laws, regulations and guidelines improved. Furthermore, efforts have been made to expedite the development of the science and technology industry, through the planning for special themes for the industry and by improving the institutional mechanism and policy environment conducive to the development of translational science and technology. The endeavours have also been made to facilitate professional and market-oriented development of the Meetings, Incentives, Conventions and Exhibitions (MICE) industry, through enhancing the professionalisation of and the solicitation of investment in the MICE industry, building a trans-regional MICE Platform, and strengthening the linkage effect of the MICE industry in attracting investment and boosting related economic sectors.

#### Other social aspects

During the pandemic, the Macau SAR government has taken proper measures to provide accommodation and work-for-relief schemes for non-resident workers 27. In the education sector, class suspension and resumption and other contingency anti-pandemic measures have been announced in a timely manner for kindergartens, non-tertiary schools and tertiary institutions, with online learning being actively promoted and optimised 28. In the meanwhile, to provide support to cross-border schoolchildren who need to pass through customs daily, the “student-only channels” were set up at the new Qingmao Port Checkpoint 29. In the public transport sector, the SAR government has worked closely with the various service providers in the marine, land and air public transport systems to implement anti-pandemic measures including bus schedule modification, capacity limits applied to public facilities in line with the situation and so on.

The Macau SAR government operates round the clock, in its various endeavours to allocate resources, make timely adjustments to anti-pandemic strategies and update relevant policies. The Macau SAR government holds regular news release conferences at which representatives of related government departments give briefings on the current pandemic situation and the prevention and control measures and explain the various pandemic control policies adopted by the government. With regard to hot issues of social concern, ad hoc representatives of relevant government departments have been invited to answer the reporters' questions directly and openly, in a bid to improve information transparency 30.

A “Special Webpage Against Pandemics” has also been set up, using infographic posters to disseminate the latest news on the pandemic situation, preventive measures, and vaccination information to residents through multiple channels, along with special webpages for NAT and vaccination appointment systems 5. At the same time, the SAR government has established cooperation mechanisms with media and telecommunications operators to ensure accurate and transparent information dissemination to the general public in a timely manner. In addition, a 24-hour hotline and a psychological support hotline have been set up to promptly handle cases 30.

The SAR government has always been committed to promoting smart healthcare in Macau. Multiple electronic systems have been established including the Macau Health Code, COVID-19 Vaccination Appointment System, Mask Sales network, telemedicine video consultation, regular and community-wide NAT booking systems and so on. The intensified application of information technology in COVID-19 pandemic prevention and control has yielded satisfactory results.

#### Cooperation with non-government bodies

The Macau SAR government invited an expert group of the National Health Commission to visit Macau, for sharing experience in pandemic prevention and control to improve the ability in and raise the level of precision prevention and control. With reference to the opinions of the expert group and accounting for the actual situation in Macau, the SAR government has continually improved and consolidated the current public health system for prevention and control in Macau.

The Macau SAR government plays a coordinating role in maintaining effective government-community cooperation. Through overall planning and effective coordination, the Macau SAR government, within a short time, mobilised the power of civil societies and community service networks to assist the SAR government in implementing various pandemic prevention measures. Here are some examples. A special enrolment page for the anti-pandemic Support Volunteer Reserve Team was established, and social workers and other professionals have been arranged to give residents psychological counselling. Furthermore, in order to increase the proportion residents to receive vaccination incentives were introduced, lottery draws were held for vaccinees to draw prizes provided by enterprises 31, 32. In addition, enterprises provided paid leaves for employees who were vaccinated, to help the government to increase vaccination rate 33. Moreover, mass media, such as Macau Daily, provided public education on COVID-19 vaccination and encouraged the public, particularly vulnerable populations including children and elderly, to get vaccination 34-37.

### The challenges in the anti-pandemic tasks in Macau

With a total population of 682,500 and a population density of 20,700 people per square kilometre 6, Macau is the most densely populated international tourist city in the world. The tourism industry is an important economic pillar in Macau, with annual visitors approaching 40 million before the COVID-19 pandemic 38, and with a recorded peak of 600,000 visits on a single day 39. The high population density and high mobility of people in Macau have made the prevention of this global COVID-19 pandemic particularly arduous.

**Figure 2:**
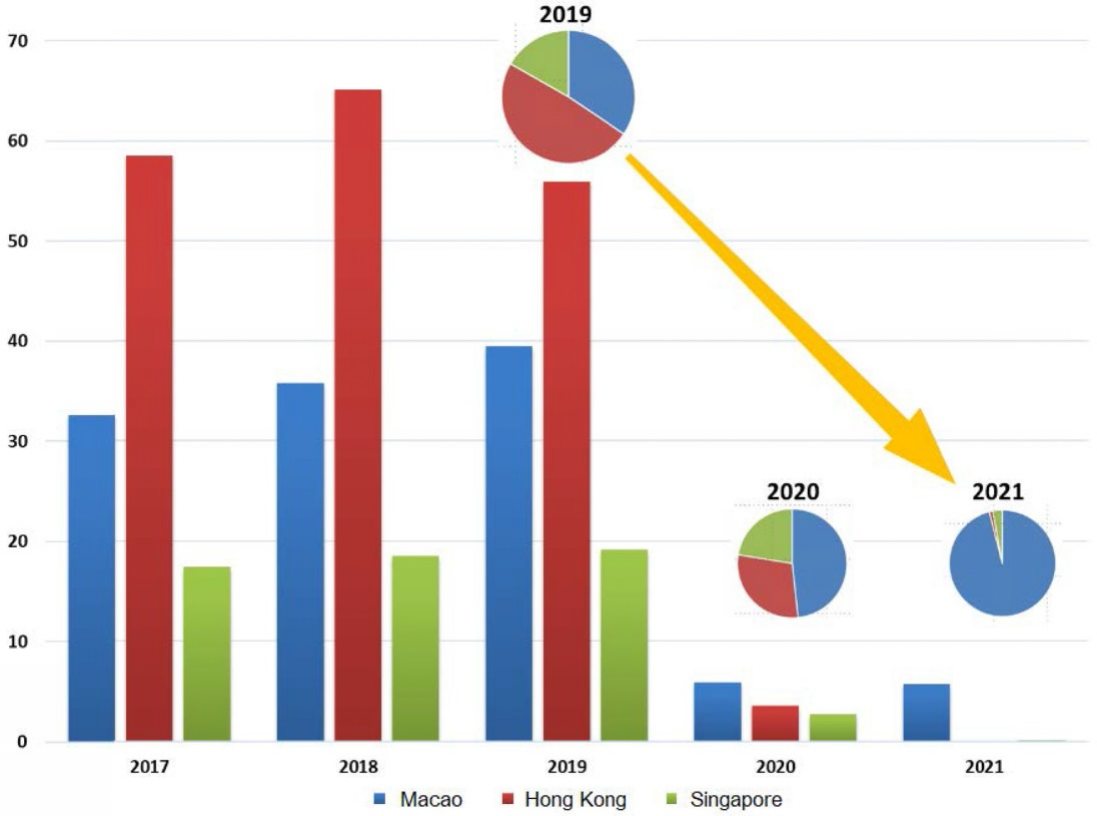
The numbers of inbound visitors to Macau, Hong Kong and Singapore 2017-2021 (Unit: million; Data source: References 7, 40, 51)

From 2020 to 2021, Macau's tourism industry has been severely affected by the pandemic. As can be seen in Figure 2, the number of visitors to Macau in 2020 dropped by nearly 90%, compared to the pre-pandemic period. As of the third quarter of 2021, the number of visitors to Macau was remained the same for the whole year of 2020, remaining at around 5 million 7. Since the outbreak of the pandemic, the number of visitors to Macau has been maintained a stable level of increasement that closely correlated to the various measures taken by the Macau SAR Government and the successful management and control of the pandemic within the region.

**Figure 3:**
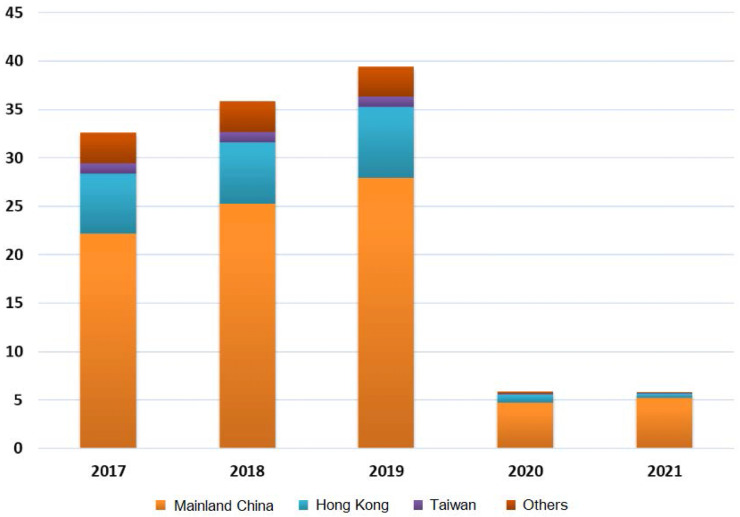
Composition of Macau's inbound visitors by origin from 2017 to 2021 (Unit: million; Data source: Reference 7)

Most of the visitors travelling to and from Macau were those from mainland China, Hong Kong and Taiwan region. As shown in Figure 3, after the outbreak of COVID-19, the majority of the visitors were still from mainland China, followed by Hong Kong, but there was a sudden drop in visitors from Taiwan region and other countries and regions 7, 39, 40. The number of visitors from certain countries and regions, such as India and Japan, as of the third quarter of 2021, dropped to tens or even single digits. The continued stabilisation of visitor numbers from mainland China results from the effective monitoring of the pandemic in both mainland China and Macau. In June 2020, Macau gradually resumed cross-border travel with Guangdong province and other provinces in the mainland, leading to a much more speedy recovery of Macau's tourism industry compared to other regions 7.

### The application of novel electronic workflows

To facilitate the speedy implementation of anti-pandemic measures and government-community collaboration for better efficiency and outcomes, digital workflows and several electronic appointment systems were adopted. As per statistics, the Macau SAR currently has following main types of digital systems, classified by their functions; the Macau Health Code System 41, the COVID-19 vaccination appointment system 42, the Nucleic Acid Test system 43, the Port and Entry/Exit Quarantine system, the Medical and Other Supporting systems 5. These systems have contributed to the application of technology and innovation.

#### The Macau Health Code (MHC) system

The Health Bureau of the Macau SAR started to develop the MHC system in April 2020, which was launched on 3 May of the same year 41, 44. The system functions covered the declarations of personal health status, travel/residence history, and contact history and combined with the Health Bureau's backend operations, assigning Red, Yellow or Green codes to their holders according to the different levels of infection risks 41. MHC also displays information such as users' nucleic acid test (NAT) dates and results issued by the testing agencies recognised by the Macau SAR and their COVID-19 vaccination records. A seamless connection with Macau's e-gates has also been established, automating part of the pandemic control procedures and reducing tremendous manual procedures and the workload of border control officers. In July 2020, a mutual recognition arrangement was made between MHC, the Guangdong Health Code of Guangdong province and the Hong Kong Health Declaration Form, creating favourable conditions for safe cross-border travel and the resumption of work, business and school between the three zones 45. The MHC system has undergone numerous improvements and optimisations according to the actual circumstances.

The Personal Data Protection Act of Macau legislation does not permit the government, an organisation or an individual to use mobile phones' signals for personal location tracking. However, the health codes of China are not subject to the same stipulation. To make travel, business and trading between the Macau SAR and mainland China possible, the seamless link between MHC and the health codes of mainland China became the priority and one of the technical challenges in the system development process. To overcome the challenge, the current MHC system matches data of users' declarations of their health status, residence/travel history, contact history with the Health Bureau's backend databases for operations and generating colour codes. This method has protected residents' privacy and made possible the seamless connection with the health codes of mainland China while complying with the regulatory requirements of the Macau SAR and mainland China 46.

#### COVID-19 Vaccination system

The COVID-19 Vaccination system was developed by the Macau SAR to support its combat with the pandemic. While it is an important means for pandemic prevention, it is also indispensable to the resumption of travel among residents between Macau and Guangdong. The system satisfies a number of requirements - that is, the validity, reliability and confidentiality of data, and the stability and high availability of the system - and the capacity to provide service on a 24-hour basis. As to its application in pandemic control and prevention, by providing reliable, stable, highly accessible instant vaccination data to various anti-pandemic system data interfaces, the system supplies powerful backend data support for the SAR's anti-pandemic work. It provides strong support for and guarantees the smooth completion of the anti-pandemic work, which constantly changes to suit the evolving pandemic situation, meeting the goal of more precise, faster, and more efficient pandemic prevention and control.

#### Nucleic Acid Test (NAT) system

The Community-wide NAT system is a user-friendly, easy-to-use appointment system, with which residents simply can input details of their identity documents, preferred location for sample collection and appointment time. The backend of the system pre-sets appointment quota available for each 30-minute slot according to the capacity of the sampling location and the number of sampling stations each location has. The quota is adjustable to enable reducing the time residents need to spend on queuing, minimising crowd gathering and the risk of cross infection.

#### Port and Exit/Entry Quarantine systems

The Macau SAR has implemented five Port and Exit/Entry Quarantine Systems: Entry Health Declaration System, Medical Observation Registration System (Front-end) and the Back-end Management System, Application System for Entry to Zhuhai with Exemption of Quarantine for Medical Observation, Customs Clearance Reservation System for Private Cars with Both Guangdong and Macau Registration Plates, and Application System for COVID-19 Entry Restriction Exemption for Foreign Nationals 47. Among these systems, the Entry Health Declaration System was integrated into MHC and replaced by it. The systems mentioned above have greatly simplified the exit/entry procedures, facilitating the efficient management and the screening of inbound and outbound travellers by new technologies. They can serve as a reference to other countries and regions and offer valuable practical experience.

#### Medical and other supporting systems

These systems include Pandemic-Related Internal Management Dashboard, Online Video / Telephone Clinic System, and Medical Voucher System with a New Function on Special Subsidies. The abovementioned systems serve to: provide the real-time enquiry of the situation in the emergency isolation area, the occupancy rate of negative pressure beds, the conditions of patients in isolation wards; review the facemask sales and inventory statistics of the health centres, contracted pharmacies and other organisations designated for the distribution of facemasks; analyse and review the inventory of personal protective supplies and drugs and provide an estimate on how long the inventory would last; enable specialists to make a diagnosis and prescribe treatments through video or phone calls; alleviate residents' medical expenses following the pandemic; contribute to the balanced development of public and private healthcare systems in Macau.

### Significant achievements in COVID-19 management

#### Effective safeguard of people's life and health

As of 11 August 2022, Macau has registered a total of 791 confirmed cases, among which only 6 death cases were reported 5. Given the population of 682,500 in Macau 6, the prevalence of COVID-19 in Macau has been relatively very low compared to other regions. With no sign of continued community transmission, Macau residents and others working and studying in this city are freed from being severely affected by the pandemic 48, 49. Of all the registered cases, 10 were reported between 22 January and 4 February 2020, with one imported-related case and the others being imported cases 11; 35 were reported between 13 March and 8 April 2020, with one imported-related case and the other 34 being imported cases; and 15 were reported between 9 April and 2 August 2021, all being imported cases^49^. From 3 August to 9 October 2021, there were two events of local spread caused by imported Delta cases, involving 4 cases and 13 cases respectively 50.

#### Macau's technical support to African countries

Macau also provided technical assistance to other countries and regions in a more direct manner. With the support of the National Health Commission, five members of the China International Medical Emergency Team (Macau) headed to Algeria and Sudan to provide emergency assistance and share their knowledge and experience in fighting against the pandemic. Their medical assistance was reported by various domestic and international media platforms. The reports also allowed other countries and regions at home and abroad to gain knowledge of Macau's pandemic management in terms of innovations, experiences, and lessons, and to adjust according to their actual situations, in the hope of drawing applications for different scenarios and regions.

### The reference significance of Macau for other countries or regions

#### The reference significance of Macau border checkpoint management for similar border checkpoints in other countries

The border checkpoint at the Gongbei Port between mainland China and Macau is one of the busiest land border checkpoints in the world (more than 134 million travellers crossed the border in 2018) 7, and the border management policies implemented by the Macau SAR government during the COVID-19 pandemic have enabled the safe and effective resumption of passenger traffic. The policies may serve as a useful reference for border management in similar ports, for example, the Woodlands Checkpoint between Singapore and Malaysia (which receives a similar number of passengers to that of the checkpoint at the Gongbei Port in Macau) 7, 51, and also the one between Gibraltar and Spain. Similarly, the guidelines developed in Macau to facilitate the safe resumption of gaming activities in casinos can also be applied to other cities with gaming facilities.

#### Reference values in Macau's management system

The Macau SAR government has developed a comprehensive management system in response to the pandemic, which contains various elements that are worth noting and referencing. In particular, the blockchain-based health code system enables health information to be transmitted and verified effectively and conveniently, while ensuring the privacy of its users by encryption. In addition, the Response and Coordination Centre for Novel Coronavirus Infection (RCC), specifically established in responding to the outbreak of the pandemic, has played a central role in providing services, enhancing community solidarity, and carrying out effective public management. At an early stage when there was limited information, the Macau government implemented resolute measures to protect the health of Macau residents and took unprecedented actions, deploying human and financial resources. It has also, by stipulating guidelines on disease prevention and risk management and utilising cutting-edge digital technologies, facilitated timely resumption of public services and economic activities. Moreover, through extensive publicity, Macau residents have been guided to cooperate with the government's anti-pandemic measures and their compliance with mask-wearing have been improved. These are what the Macau government has achieved in its course of fighting against the pandemic, the experiences and evidence that can serve as a useful reference for other countries and regions.

#### Reference values of MHC for other regions with close cross-border exchanges

Because of the differences in the laws and regulations on personal data protection in Macau and mainland China, the development and application of the Health Codes of the two regions were different. However, given the long-standing exchanges, cooperation and movement of persons between the two regions and the need to ensure that the exchanges, cooperation, trades and tourism activities of the two regions could carry on smoothly, the Macau SAR developed and built independently its first Health Code System on the basis of the users' declarations of health status, travel/residence history, contact history and the various relevant backend databases of the Macau Health Bureau. The system not only meets the regulatory requirements for personal data protection in Macau, but also seamlessly connects with the mainland health codes, laying an important foundation for exchanges and cooperation between the two regions. Other regions with close exchanges with another region whose regulatory requirements are different can take the experience of the Macau Health Code as a reference for inspirations and viable solutions.

### The latest wave of the COVID-19 outbreak and immediate response in Macau

In June 2022, a new wave of COVID-19 outbreak occurred in Macau due to invisible transmission in the community. During the period from June 18, 2022 to August 11, 2022, the Health Bureau of Macau SAR government has registered 708 new confirmed cases and 1,259 new asymptomatic infection cases 5. From June 19, 2020 when the Chief Executive of Macau SAR announced that Macau entered “the state of immediate prevention in relation to COVID-19” 52 to August 11, 2022, Macau government has successfully controlled this wave of outbreak, and then reopened the border gate with mainland China on August 3, 2022 53. This is a great achievement because apart from mainland China, Macau is the only area that successfully controlled the COVID-19 outbreak caused by the Omicron BA.5, the currently most contagious SARS-CoV-2 54, without adopting the “lockdown” policy.

To prevent the spread of the virus, Macau SAR government took several new actions immediately.

#### Temporary shut-down of borders with mainland China

The Macau SAR government has decided to temporarily shut down the borders with Zhuhai since 23 June 2022, while all passengers from Macau to Zhuhai are required to take 7-day quarantine 55. This measure could effectively prevent the spread of contagion in Macau, and control the risk of transmission within a relatively small area, reducing the difficulty in controlling the current outbreak.

#### Immediate nucleic acid tests for all population

Since the current outbreak in Macau (i.e., 18 June 2022) 56 to 11 August 2022, Macau SAR government has planned to organize 14 rounds of all population nucleic acid testing 57-61. Repetitive large-scale nucleic acid testing could timely identify infected cases, facilitating subsequent epidemiological surveys, close contact tracking, concentrated medical observation, medical isolation and treatment 62.

#### Free distribution of KN95 masks and self-test COVID-19 antigen kits

Macau SAR government has distributed KN95 masks and self-test COVID-19 antigen kits to all people in Macau, including permanent residents, foreign employees, or cross-border students 63. Self-test COVID-19 antigen kits could effectively and timely screen infected cases and reduce the risk of cross-infection between people who received large-scale nucleic acid tests. In addition, the policy of distributing KN95 masks and self-test COVID-19 antigen kits without distinction reflects Macau SAR government's commitment of “leave no one behind” 64.

## Discussion

The Macau SAR government has applied innovative and advanced technologies. Simultaneously, it has actively coordinated and developed partnerships with non-governmental bodies. The COVID-19 prevention and control results are remarkable. The efforts have effectively protected the life, health and safety of the people. The measures used are sustainable and can serve as a significantly important reference for other countries/regions, which are in line with the “Global Development Cooperation” goal in the UN Millennium Development Goals 65.

The anti-pandemic measures in Macau are almost consistent with the "dynamic zero-COVID policy" in mainland China, with some appropriate adaptions according to the local socioeconomic contexts. The close connection between Macau and mainland China is a contributing factor to the low prevalence of COVID-19 and also the great achievement of COVID-19 management in Macau, which helps Macau to keep the openness of border between Macau and mainland China, and the large amount of tourist flow from mainland China is helpful to sustain the economic development of Macau 66, 67.

Generally speaking, the measures and methods for managing COVID-19 in the Macau region have almost reached perfection, with the only shortfall that the economy has not yet recovered to its level as of before the pandemic outbreak because the economic connections between Macau and other countries are almost at a standstill. In view of this, since the consumption volume of local residents in Macau is relatively small and its economic development relies much on tourism and service industries, it is recommended that the Macau SAR government can make reference to the model of the development of the Internet economy of mainland China and vigorously develop e-commerce, Internet shopping, video clip marketing and so on. The government can also coordinate and assist in terms of payment, logistics, and management, in order to increase the size of Macau's economy and facilitate the rapid recovery and development of the economy.
